# Cationic nano-copolymers mediated *IKKβ* targeting siRNA inhibit the proliferation of human Tenon’s capsule fibroblasts in vitro

**Published:** 2008-12-31

**Authors:** Yongheng Duan, Xipeng Guan, Jian Ge, Daping Quan, Yehong Zhuo, Hehua Ye, Tingting Shao

**Affiliations:** 1 State Key Laboratory of Ophthalmology, Zhongshan Ophthalmic Center, Sun Yat-sen University, Guangzhou, China; 2 Institute of Polymer Science, School of Chemistry and Chemical Engineering, Sun Yat-sen University, Guangzhou, China

## Abstract

**Purpose:**

To synthesize a ternary cationic copolymer called CS-*g*-(PEI-*b*-mPEG) and characterize its features as a non-viral siRNA carrier; in turn, to investigate the influence of small interfering RNA (siRNA) targeting IκB kinase subunit β (*IKKβ*) on the proliferation of human Tenon’s capsule fibroblasts (HTFs) in vitro.

**Methods:**

First, a novel cationic copolymer composed of low molecular weight, linear poly(ethyleneimine) [PEI] blocked with polyethylene glycol (PEG) and grafted onto a chitosan (CS) molecule was synthesized. CS-*g*-(PEI-*b*-mPEG) was then compacted with 21nt siRNA at various copolymer/siRNA charge (N/P) ratios, and the resulting complexes were characterized by dynamic light scattering, gel electrophoresis, and serum incubation. Cell Titer 96^®^ AQ_ueous_ One Solution cell proliferation assay was used to investigate the cytotoxicity of this cationic copolymer. Second, siRNAs targeting *IKKβ* (IKKΒ-siRNAs) were delivered into the HTFs using CS-*g*-(PEI-*b*-mPEG) as the vehicle. Real-time reverse transcription polymerase chain reaction (RT–PCR) subsequently assessed the mRNA level of *IKKβ*, and western blot assay was used to determine protein expression. After IKKB-siRNA transfection, Cell Titer 96^®^ AQ_ueous_ One Solution cell proliferation assay was used to evaluate the proliferation of HTFs.

**Results:**

The diameter of the CS-*g*-(PEI-*b*-mPEG)/siRNA complexes tended to decrease whereas their zeta potential tended to increase as the N/P ratio increased. The CS-*g*-(PEI-*b*-mPEG) copolymer showed good siRNA binding ability and high siRNA protection capacity. Furthermore, the copolymer presented remarkable transfection efficiency and showed much less cytotoxicity than 25 kDa PEI. IKKB-siRNAs were successfully delivered into HTFs using CS-*g*-(PEI-*b*-mPEG) as a vector. As a result, the expression of *IKKβ* was downregulated at both the mRNA and protein levels, and the activation of nuclear factor-κB (NF-κB) in the HTFs was subsequently inhibited. Most impressively, the proliferation of HTFs was also effectively suppressed through the blocking of the NF-κB pathway.

**Conclusions:**

All the results demonstrate that CS-*g*-(PEI-*b*-mPEG) is a promising candidate for siRNA delivery, featuring excellent biocompatibility, biodegradability, and transfection efficiency. The RNA interference (RNAi) strategy using cationic copolymers as siRNA carriers will be a safe and efficient anti-scarring method following glaucoma filtration surgery.

## Introduction

RNA interference (RNAi) was originally recognized as an evolutionary conserved defense mechanism in higher eukaryotic cells, and this system can easily and effectively inhibit the expression of one specific gene [1]. The RNAi process is mediated through small, double-stranded RNA molecules called small interfering RNA (siRNA), which specifically trigger the cleavage and subsequent degradation of their target mRNA in a sequence-dependence manner. Hence, synthesis of the protein encoded by those mRNA is prevented [2]. Recently, RNAi-mediated gene silencing has also been shown to be efficient in mammalian cells, and this has led to the increasing feasibility of RNAi technology for the therapy of certain human diseases [3].

The efficiency of RNAi mainly depends on the successful delivery of intact siRNA into mammalian cells. However, due to the low stability of siRNA against enzymatic degradation and low permeability across cell membranes, the efficacy of naked siRNA is insufficient. Therefore, it is necessary to develop efficient and convenient methods for siRNA delivery. Until now, viral delivery systems have been used as vectors for genes in many studies due to the advantage of high transfection efficacy, but the use of such delivery systems is limited by endogenous recombination and host immunity [4]. Moreover, since ontogenesis and mortality have been reported [5], concerns have been raised regarding the safety of using viral vectors in gene therapy trials in humans. In light of these problems, studies of alternative delivery strategies focus on non-viral systems for gene delivery. Recently, cationic copolymers have been demonstrated to be a promising non-viral vector for transfecting nucleotides into various cell types or tissues [6]. Among these cationic polymers, polyethylenimine (PEI) is an effective gene carrier due to PEI’s high charge density and endosomal disruption function, but it is difficult to achieve both the goals of higher transfection efficiency and lower cytotoxicity with PEI homopolymers [7]. Various modifications of PEI have been investigated to promote its nucleotide delivery ability as well as to reduce its adverse effects on cell viability. It has been shown that grafting PEI with nonionic hydrophilic polymers such as polyethylene glycol (PEG) could be an effective approach for minimizing the cationic toxicity of PEI and that the cationic toxicity of PEI decreases as its molecular weight decreases [8]. However, the transfection efficiency of the PEGylated, low molecular weight PEI copolymer is lower than that of 25 kDa PEI [9]. Chitosan (CS) is a non-toxic, biodegradable cationic polymer with relatively low immunogenicity and especially good macro-adhesion. CS has been extensively investigated as a delivery system for therapeutic macromolecules, nucleotides, and protein molecules [10]. Thus, we hypothesize that a new copolymer could be synthesized as a siRNA carrier that would have both the efficient transfection ability of PEI and the biocompatibility of PEG and CS.

Glaucoma is an eye disease usually associated with increased intraocular pressure that leads to irreversible functional impairment of the optic nerve. Filtration surgery to enhance the drainage of aqueous humor is one of the most effective therapies for glaucoma [11], but the therapy’s success rate is reduced by blockage of the surgically created drainage channel by subconjunctival scarring that may occur with wound healing [12]. Fibroblasts located in the subconjunctival area play a major role in scar formation after filtration surgery through proliferation, migration, and synthesis of the extracellular matrix (ECM). Thus, regulating the biological activities of subconjunctival Tenon’s capsule fibroblasts (TCFs) during the wound healing process is a major anti-scarring strategy for glaucoma filtration surgery [13]. Nuclear factor-κB (NF-κB) is a transcription factor that is also a positive regulator for fibroblasts, and a protein complex called IκB kinase (IKK) is a critical regulator of the activation of NF-κB [14,15]. Most studies on the role of the NF-κB pathway in the regulation of cell proliferation have used immortal cell lines (cells capable of continuously renewing themselves). In this study, we investigated whether inhibiting the function of IKK and subsequently blocking the signaling pathway of NF-κB could effectively manipulate the activation and proliferation of TCFs during the scarring process following glaucoma filtration surgery.

In the study reported here, absorbable, low molecular weight PEI was blocked with polyethylene glycol monomethyl ether (mPEG) and grafted onto chitosan. As a result, a novel biodegradable copolymer, CS-*g*-(PEI-*b*-mPEG), was synthesized. The properties of the CS-*g*-(PEI-*b*-mPEG)/siRNA complexes such as particle size, zeta potential, siRNA binding and protection capacity, transfection ability, and cytotoxicity were studied, and IKKΒ-siRNAs were then delivered into human Tenon’s capsule fibroblasts (HTFs) using CS-*g*-(PEI-*b*-mPEG) as the vehicle. The expression of IKKβ was detected at both the mRNA and protein levels after the transfection of IKKΒ-siRNAs. We also investigated the activation of NF-κB and the proliferation of HTFs after the RNA interference process targeting *IKKβ*.

## Methods

### Cell culture

HeLa (human cervix epithelial carcinoma) cells were obtained from the American Type Culture Collection (Number CCL-2.1; ATCC, Rockville, MD) and were maintained in Dulbecco’s modified Eagle’s medium (DMEM; Gibco, Grand Island, NY) containing 10% fetal bovine serum (FBS; HyClone, Logan, UT), 2 mM of L-glutamine, 100 IU/ml of penicillin, 100 μg/ml of streptomycin, and 25 μg/ml of amphotericin B (all from Sigma-Aldrich, St. Louis, MO) at 37 °C with 5% CO_2_, 95% humidified atmosphere.

Tissue explants of human Tenon’s capsule were obtained from three male patients (aged 28, 39, and 62 without any topical eye treatment) who had undergone trauma or cataract surgery. Patients were informed of the nature and possible consequences of the tissue removal procedure, and written, informed consent was obtained. The tenets of the Declaration of Helsinki were followed, and approval by the Sun Yat-sen University Human Experimentation committee was granted. HTFs were cultured by a previously reported method [16] with some modification, as described below. Cells were maintained as a monolayer at 37 °C with 5% CO_2_, 95% humidified atmosphere in DMEM supplemented with 10% FBS, 2 mM of L-glutamine, 100 IU/ml of penicillin, 100 μg/ml of streptomycin, and 25 μg/ml of amphotericin B. Cells between passages 3 and 6 were used for the following experiments.

### Synthesis and characterization of CS-*g*-(PEI-*b*-mPEG)

**Figure 1:**
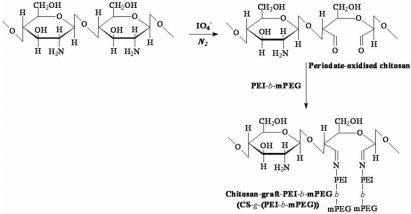
The synthesis schedule of CS-g-(PEI-b-mPEG) copolymer.

Chitosan, (the weight-average molecular weight of chitosan was 3.50×10^5^ [Mw=3.50×10^5^], which was measured by the viscosity method and the degree of deacetylation of chitosan was 88%, which was determined by proton nuclear magnetic resonance [^1^H NMR]), was purified by a solvent precipitation method. Polyethylene glycol monomethyl ether (mPEG, AR, M_n_=2000) was purchased from Sigma-Aldrich Chemie Gmbh (Steinheim, Germany). Linear PEI (M_n_=600) and branched PEI (M_n_=25,000) were obtained from Wako Pure Chemical Industries, Ltd. (Osaka, Japan). CS-*g*-(PEI-*b*-mPEG) was synthesized by Jiang’s method with some modification [17], and the steps are briefly described as follows. Di-block copolymer, PEI-*b*-mPEG, was synthesized by an imine reaction, and then the periodate ion, IO_4_^-^, was used to oxidize CS to produce dialdehyde. A novel, comb-like copolymer, CS-*g*-(PEI-*b*-mPEG), was synthesized by an imine reaction between the amino groups of PEI-*b*-mPEG and the aldehyde groups of periodate-oxidized CS. The resultant product was purified by dialysis against double deionized (DD) water with the use of Spectra/Pro2 membrane (molecular weight cut off was 12 K; Spectrum, Houston, TX) for 72 h to remove the unreacted PEI and mPEG and then freeze-dried for another 24 h. A schematic illustration of the synthesis process is shown in Figure 1, and the structure of the newly synthesized CS-*g*-(PEI-*b*-mPEG) was proved by ^1^H NMR and gel permeation chromatography (GPC).

### Preparation of CS-*g*-(PEI-*b*-mPEG)/siRNA complexes

IKKΒ-siRNA, scrambled siRNA, and fluorescein isothiocyanate (FITC)-conjugated scrambled siRNA were all synthesized by Ribobio Co. Ltd. (Guangzhou, China). Lyophilized siRNAs were dissolved in RNase-free H_2_O (pH=7.4) and incubated for 5 min at room temperature, and the CS-*g*-(PEI-*b*-mPEG) copolymer was dissolved in serum-free DMEM at a stock concentration of 1 mg/ml. Subsequently, different concentrations of the CS-*g*-(PEI-*b*-mPEG) solution were added into dissolved siRNA to make complexes of various charge ratios. The charge ratio of CS-*g*-(PEI-*b*-mPEG) and siRNA was expressed as the molar ratio of the amine groups of the copolymer (representing positive units) to the phosphates of siRNA (representing negative units) called the N/P ratio. The mixture was gently vortexed for 10 s and incubated at room temperature for 30 min to allow the formation of CS-*g*-(PEI-*b*-mPEG)/siRNA complexes.

### Measurement of particle size and zeta potential

Dynamic light scattering (DLS) with a Zetasizer Nano ZS instrument (Malvern Instruments, Worcestershire, UK) was used to measure the diameter and zeta potential of the CS-*g*-(PEI-*b*-mPEG)/siRNA complexes with N/P ratios of 1, 3, 5, 10, and 20. The hydrodynamic diameter of the freshly prepared complexes was measured at 25 °C with a scattering angle of 90° (10 mW He-Ne Laser, 633 nm), and the zeta potential was determined by the standard capillary electrophoresis cell of Zetasizer Nano ZS at position 17.0 and at 25 °C. All the average values were performed with the data from three separate measurements.

### Electrophoresis mobility assay

The binding degree between CS-*g*-(PEI-*b*-mPEG) and siRNA was determined by 4% agarose (low melting point) gel electrophoresis [10]. The complexes were prepared at N/P ratios of 0 (samples containing siRNAs that did not compact with CS-*g*-(PEI-*b*-mPEG) were considered as N/P ratio=0), 1, 3, 5, 10, and 20 as described above, and samples, each containing 0.133 μg (1×10^−2^ nmol) of siRNAs, were loaded onto 4% agarose gel together with 1:6 dilution of the loading buffer. The electrophoresis was performed in TBE buffer (4.5 mM of Tris-base, 1 mM of sodium EDTA, 4.5 mM of boric acid, pH=8.3) at 55 V for 1 h. To visualize the siRNA, the gel was immersed in 0.5 µg/ml of ethidium bromide solution (Sigma-Aldrich), and the fluorescence images were captured under ultraviolet (UV) illumination (Vilber Lourmat, France).

### Serum resistance test

The ability of CS-*g*-(PEI-*b*-mPEG) to protect siRNA against enzymatic degradation was investigated according to a previously reported method with minor modifications [10]. The CS-*g*-(PEI-*b*-mPEG)/siRNA complexes containing the same amount of siRNAs were prepared at an N/P ratio of 0 (naked siRNA), 1, 3, 5, 10, and 20. Subsequently, FBS was added as needed to achieve a final concentration of 10%. The mixtures were incubated at 37 °C, and at each determined time interval (2 h, 4 h, 8 h, 12 h, and 24 h), a sample of each N/P ratio was removed and incubated at 70 °C for 5 min to inactivate the serum enzymes. Then, 5 µl of heparin (1000 IU/ml) was added to displace the siRNAs from the complexes before the mixtures were loaded onto a 15% polyacrylamide gel containing 7 M urea. Electrophoresis was performed in TBE buffer at 200 V for 1 h. Afterward, gels were stained in a 1:10,000 dilution of SYBR^®^ Green II fluorescent RNA dye (Molecular Probe Inc., Eugene, OR) for 40 min, and Bio-Capt version 10.0 software (Vilber Lourmat, France) was used to analyze the fluorescence intensity of each band. All experiments were performed in triplicate, and the fluorescence intensity of each band was compared with that of the non-FBS treated siRNAs (which served as the control) on the same gel.

### In vitro transfection and cell viability assays

HeLa cells and HTFs were plated in six well plates with a density of 6×10^5^ cells per well and incubated for 12 h or 24 h (reaching 60%–70% confluence). The culture media were then replaced with DMEM without serum or with antibiotics 2 h before transfection. The CS-*g*-(PEI-*b*-mPEG) and FITC-conjugated siRNA complexes were prepared as described above, and the N/P ratios were performed at 0 (naked FITC-conjugated siRNAs), 1, 3, 5, 10, and 20. A total volume of 2 ml of serum and antibiotics-free DMEM-containing complexes was added to each well, and the final concentration of siRNAs was 50 nM. After a 6 h incubation at 37 °C, the remaining media were discarded, and cells were trypsinized and resuspended in PBS at a density of 5×10^5^ cells/ml for flow cytometry analysis. The transfection efficiency was calculated by measuring the percentage of FITC-labeled cells using a FACSAria^TM^ System (BD Bioscience, Oxford, UK).

Cell Titer 96^®^ AQ_ueous_ One Solution cell proliferation assay (Promega, Madison, WI) was used to evaluate cell viability [18]. HTFs were seeded in 96 well plates with an initial density of 5×10^3^ cells per well and incubated for 24 h (reaching 80% confluence) before the copolymers were added. Then, serum-supplied DMEM were replaced by 200 µl of serum and antibiotic-free DMEM that contained various concentrations (1, 5, 10, 50, and 100 μg/ml) of CS-*g*-(PEI-*b*-mPEG) or 25 kDa PEI. The CS-*g*-(PEI-*b*-mPEG) or 25 kDa PEI/siRNA complexes containing 100 nM of siRNAs were prepared at an N/P ratio of 0 (only scrambled siRNAs), 1, 3, 5, 10, and 20, and their cytotoxicity was also evaluated. After 24 h incubation at 37 °C, the media were replaced with fresh serum and antibiotic-free DMEM that contained 20 μl of Cell Titer 96^®^ AQ_ueous_ One Solution Reagent (MTS). Finally, after 4 h of additional incubation, a micro-plate reader (Bio-Rad Lab Inc., Hercules, CA) measured the absorbance of each well at 570 nm. Cell viability was calculated according to the following equation: 

Cell viability (%)=(OD570(sample)/OD570(control)) x 100

where OD_570(sample)_ represents the average absorbance of cells treated with media that contain different concentrations of cationic polymers or cationic polymers/siRNA complexes, and OD_570(control)_ represents the average absorbance of cells treated only with an equal volume of serum-free DMEM.

### Delivery of IKKB-siRNA into human Tenon’s capsule fibroblasts via CS-*g*-(PEI-*b*-mPEG)

Two pairs of siRNA specifically targeting *IKKβ* (IKKΒ-siRNA) were derived from the coding sequence of the human *IKKβ* gene (GenBank NM_001556) and were designed using a siRNA Target Finder program. A BLAST search checked all the duplex sequences and target sequences of these siRNAs to preclude sequences with significant similarity to other genes in the human genome. The duplex sequences of IKKB-si1 were 5′-CCG ACA UUG UGG ACU UAC AdT dT, dTd TGG CUG UAA CAC CUG AAU GU-5′, and the duplex sequences of IKKB-si2 were 5′-GCU UAG AUA CCU UCA UGA AdT dT, dTd TCG AAU CUA UGG AAG UAC UU-5′. HTFs were plated in six well plates with a density of 6×10^5^ cells per well and incubated for 12 h. Subsequently, the culture media were replaced with serum- and antibiotic-free DMEM 2 h before transfection. CS-*g*-(PEI-*b*-mPEG)/IKKΒ-si1 and CS-*g*-(PEI-*b*-mPEG)/IKKΒ-si2 complexes were prepared at an N/P ratio of 10 30 min before transfection, and cells were incubated with serum- and antibiotic-free DMEM that contained complexes corresponding to the determined final concentrations of IKKΒ-si1 or IKKΒ-si2 (5, 10, 25, 50, and 100 nM) for 6 h. Then, the cells were maintained in serum-supplied DMEM for another 24 h or 48 h before the following assays were performed as described below. Non-transfected HTFs were regarded as the control, and cells were also transfected with 100 nM of scrambled siRNA.

### Real-time reverse transcription polymerase chain reaction

Total RNA was extracted from 1×10^5^ to 2×10^5^ HTFs 24 h after the transfection medium was removed using the RNeasy Micro Kit (Qiagen Inc., Valencia, CA) according to the manufacturer’s protocol. The yield and purity of the RNA were spectrophotometrically determined, and the cDNA were prepared using the ReverAid^TM^ First Strand cDNA Synthesis Kit (Fermentas Inc., Hanover, MD). A real-time reverse transcription polymerase chain reaction (RT–PCR) procedure was conducted according to the manufacturer’s protocol for the SYBR^®^ Premix Ex Taq^TM^ Kit (Takara Biotechnology, Otsu, Shiga, Japan). Reaction participants were assembled in a 96 well optical reaction plate (Applied Biosystems, Foster City, CA), and each well contained SYBR^®^ Premix Ex Taq^TM^ (2X), 200 nM of forward primer, 200 nM of reverse primer, ROX Reference Dye (50X), and cDNA solution with a total volume of 20 μl. For *IKKβ*, the forward primer was 5′-TGT CAG TGG AAG CCC GGA TAG-3′, and the reverse primer was 3′-AGG TTA TGT GCT TCA GCC ACC AG-5′. The mRNA level of glyceraldehydes-3-phosphate dehydrogenase (*GAPDH*) was also measured in each sample as an internal control. The forward primer was 5′-ATC ACC ATC TTC CAG GAG CGA-3′, and the reverse primer was 3′-CAG AAG TGG TGG TAC CTC TTC C-5′. Reactions were performed under the following conditions: 10 min at 95 °C for the initial denaturation, 40 cycles of amplification (5 s at 95 °C), and annealing for 31 s at 60 °C, using the ABI Prism 7000 Sequence Detection System (Applied Biosystems). The threshold cycle (C_t_) values were determined by ABI Prism 7000 Software (Applied Biosystems) and were normalized by subtracting the C_t_ *GAPDH* values. All experiments were performed in triplicate, and the relative amount of mRNA of each sample was calculated using the 2^-ΔCt^ method in individual experiments [19].

### Western blot

Each group of HTFs was lysed in lysis buffer (60 mM of Tris, 2% SDS, 100 mM of 2-mercaptoethanol, and 0.01% bromophenol blue) 48 h after the transfection procedure. An equal amount of protein (10 µg) was loaded on 12% sodium dodecyl sulfate-polyacrylamide gel, and electrophoresis was performed for 1 h. The proteins were then electrophoretically transferred to a polyvinylidene diflouride (PVDF) membrane (Invitrogen, Carlsbad, CA) for probing with mouse monoclonal anti-IKKβ (BD Bioscience, San Jose, CA) and horseradish peroxidase (HRP)-conjugated goat anti-mouse IgG (Santa Cruz Biotechnology Inc., Santa Cruz, CA). Blotting signals were detected by chemiluminescence reagents using an ECL kit (Amersham Bioscience, Piscataway, NJ) following the manufacturer’s instructions. The β-actin protein amount of each sample was also measured as an internal control.

### Confocal laser scanning microscopy

HTFs prepared for the confocal microscopy study were seeded onto preloaded glass coverslips (18 mm×18 mm) in six well plates with a density of 6×10^5^ cells per well and incubated for 24 h to allow adhesion. Then, 100 nM of IKKΒ-siRNA or 100 nM of scrambled siRNA were transfected into HTFs as described above, and after another 24 h, the cells were stimulated with 20 ng/ml of tumor necrosis factor-α (TNF-α) for 1 h. All the coverslips were taken out 24 h after the TNF-α stimulation and were rinsed three times with PBS. The cells were then fixed by incubation with 4% paraformaldehyde solution at room temperature for 10 min followed by 10 min permeabilization by 0.2% Triton X-100 (Sigma-Aldrich). After blocking the nonspecific binding with goat serum for 30 min, all the samples were incubated with mouse monoclonal anti-p65 of NF-κB (1:100, Santa Cruz Biotechnology Inc.) and FITC-conjugated goat anti-mouse IgG (1:200, Santa Cruz Biotechnology Inc.) at 37 °C for 1 h, both under light exclusion. The nuclei of the cells were counterstained with 2 µg/ml of DAPI (4’, 6-diamidino-2-phenylindole dihydrochloride) at room temperature for 10 min under light exclusion. A Zeiss LSM 510 confocal laser scanning device (Zeiss, Oberkochen, Germany) was used to capture the inflorescence images. Cells not treated with either CS-*g*-(PEI-*b*-mPEG)/siRNA complexes or TNF-α were considered the negative control, and cells treated with only 20 ng/ml of TNF-α for 1 h were measured as the positive control.

### Cell proliferation assay

The proliferation of HTFs was measured using Cell Titer 96^®^ AQ_ueous_ One Solution cell proliferation assay. After siRNA transfection, each group of HTFs was trypsinized and seeded in a 96 well plate with an initial density of 5×10^3^ cells per well. Subsequently, cells were cultured in a serum-supplied medium for 72 h, and the absorbance of each sample corresponding to the cell number was measured.

### Statistical analysis

All data are presented as means±standard deviation (SD). The statistical analyses were conducted using SPSS version 13.0 for windows (SPSS Science Inc., Chicago, IL). Statistical analysis was performed using Student’s *t*-test or one-way analysis of variance (ANOVA). Probability (p) of less than 0.05 was considered significant.

## Results

### The physicochemical properties of CS-*g*-(PEI-*b*-mPEG)

**Figure 2:**
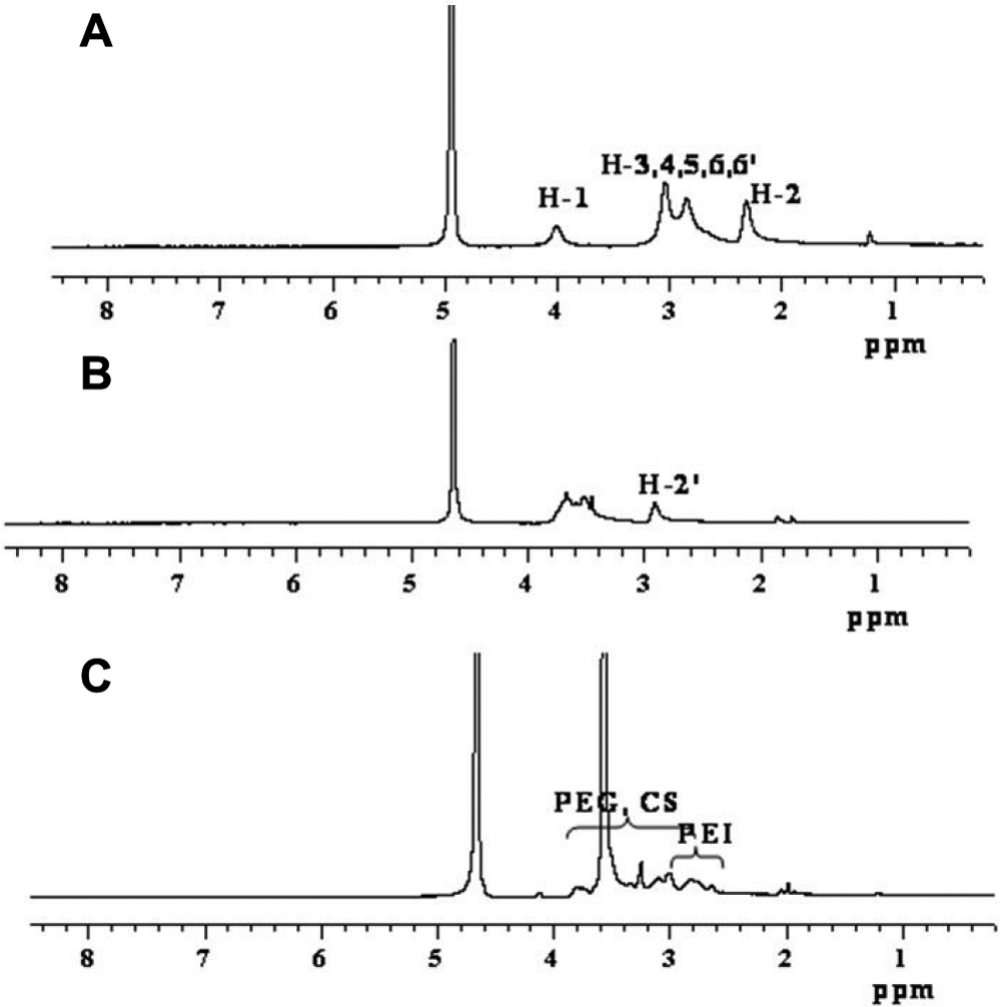
The ^1^H NMR spectra of CS and its derivatives. Spectra were obtained with 32 scans, and delay of 2 s between pulses. **A**: CS in D_2_O and CF3COOD at 293 K, **B**: CS-CHO in D_2_O at 293 K; **C**: CS-g-(PEI-b-mPEG) in D_2_O at 293 K.

CS proton signals appeared at 2.84 ppm (H-2) and 3.3−3.8 ppm (H-3, 4, 5, 6, 6’; Figure 2B), and the oxidation degree of CS (*q*) was calculated by the following equation from the ^1^H NMR spectrum:

q=(IH2-IH2')/(IH2) x 100%

From the results of GPC (Table 1), the molecular weights of periodate oxidation CS and CS-*g*-(PEI-*b*-mPEG) were 1.16×10^4^ and 2.95×10^4^, and the degree of grafted PEI-*b*-mPEG can be calculated by the following equation according to the data above:

M’_W,CS-CHO_ is the average molecular weight of repeat unit of CS-CHO.

**Table 1:** Characteristics of prepared CS-*g*-(PEI-*b*-mPEG).

Degree of oxidation (CS; mol %)*	Mw of periodate- oxidation CS (CS-CHO)**	Mw of CS-*g*-(PEI-*b*-mPEG)**	Degree of grafted PEI-*b*-mPEG (mol %)**
36	1.16×10^4^	2.95×10^4^	8.1

The asterisk means that it was determined by ^1^H NMR, and a double asterisk indicates that it was determined by GPC.

q=[(MW,CS-g-(PEI-b-mPEG)-MW,CS-CHO)/MW,PEI-b-mPEG]/(MW,CS-CHO/M'W,CS-CHO) x 100%

### Particle sizes and zeta potential of the complexes

**Figure 3:**
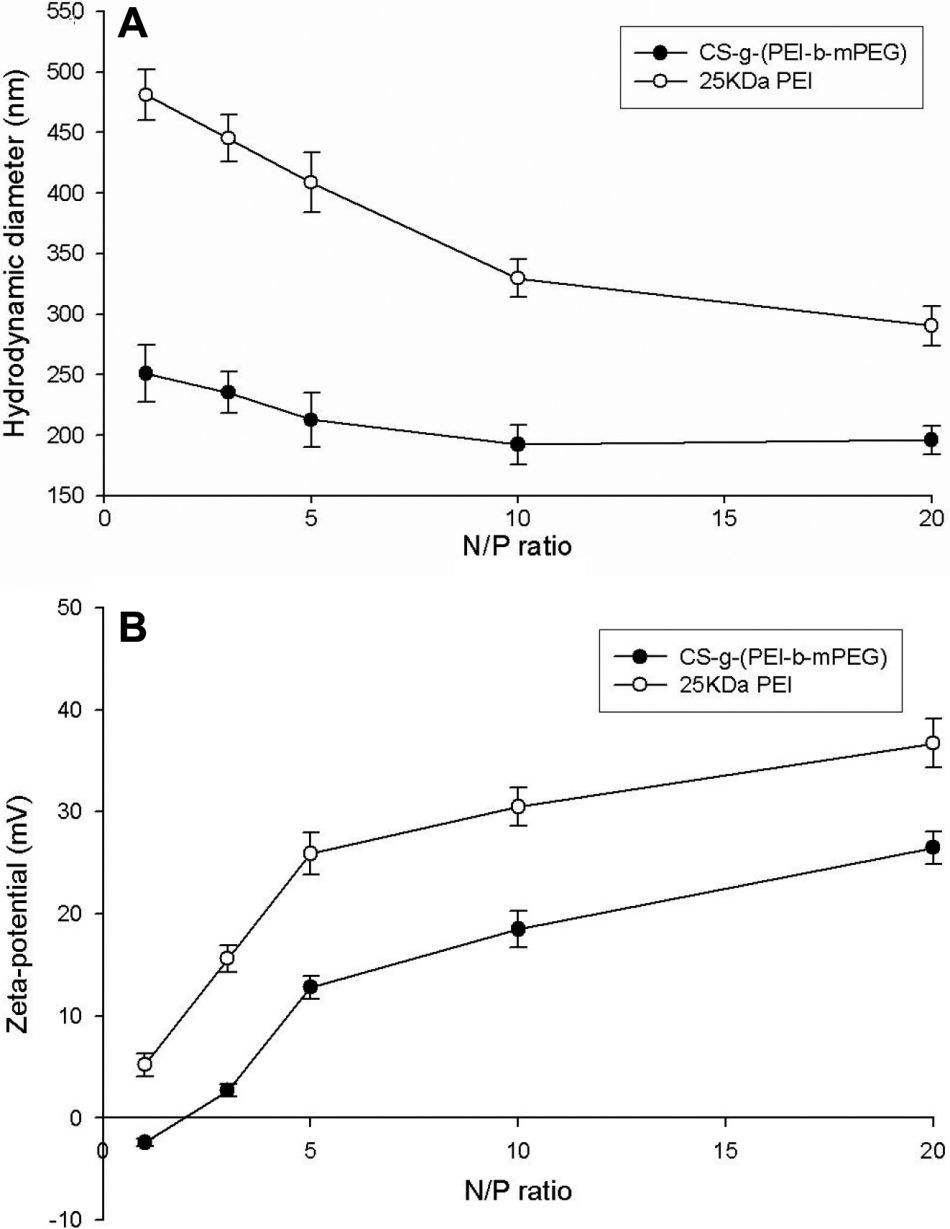
The effects of N/P ratio on the hydrodynamic diameter and zeta-potential of CS-*g*-(PEI-*b*-mPEG)/siRNA complexes and 25 kDa PEI/siRNA complexes. The diameter of complexes decreased while the N/P ratios increased from 1 to 10 and then remained constant up to N/P of 20; the zeta-potential of complexes increased in parallel with the rinsing N/P ratio. **A**: hydrodynamic diameter (mean±SD, n=3); **B**: zeta-potential (mean±SD, n=3).

The diameter of the CS-*g*-(PEI-*b*-mPEG)/siRNA complexes ranged between 200 nm and 250 nm at the determined N/P ratios (Figure 3A), which was less than that of 25 kDa PEI at each N/P ratio. It should be noted that the diameter of the complexes decreased while the N/P ratios increased from 1 to 10 and then remained constant up to an N/P ratio of 20. The zeta potential of the complexes increased in parallel with the rinsing N/P ratio, ranging from −2.4 mV to +26.5 mV. The complexes prepared at all the determined N/P ratios showed a lower zeta potential compared with that of the 25 kDa PEI/siRNA complexes, which was measured as +5.2 mV—+36.7 mV (Figure 3B).

### Condensation and protection ability of CS-*g*-(PEI-*b*-mPEG) for siRNA

**Figure 4:**
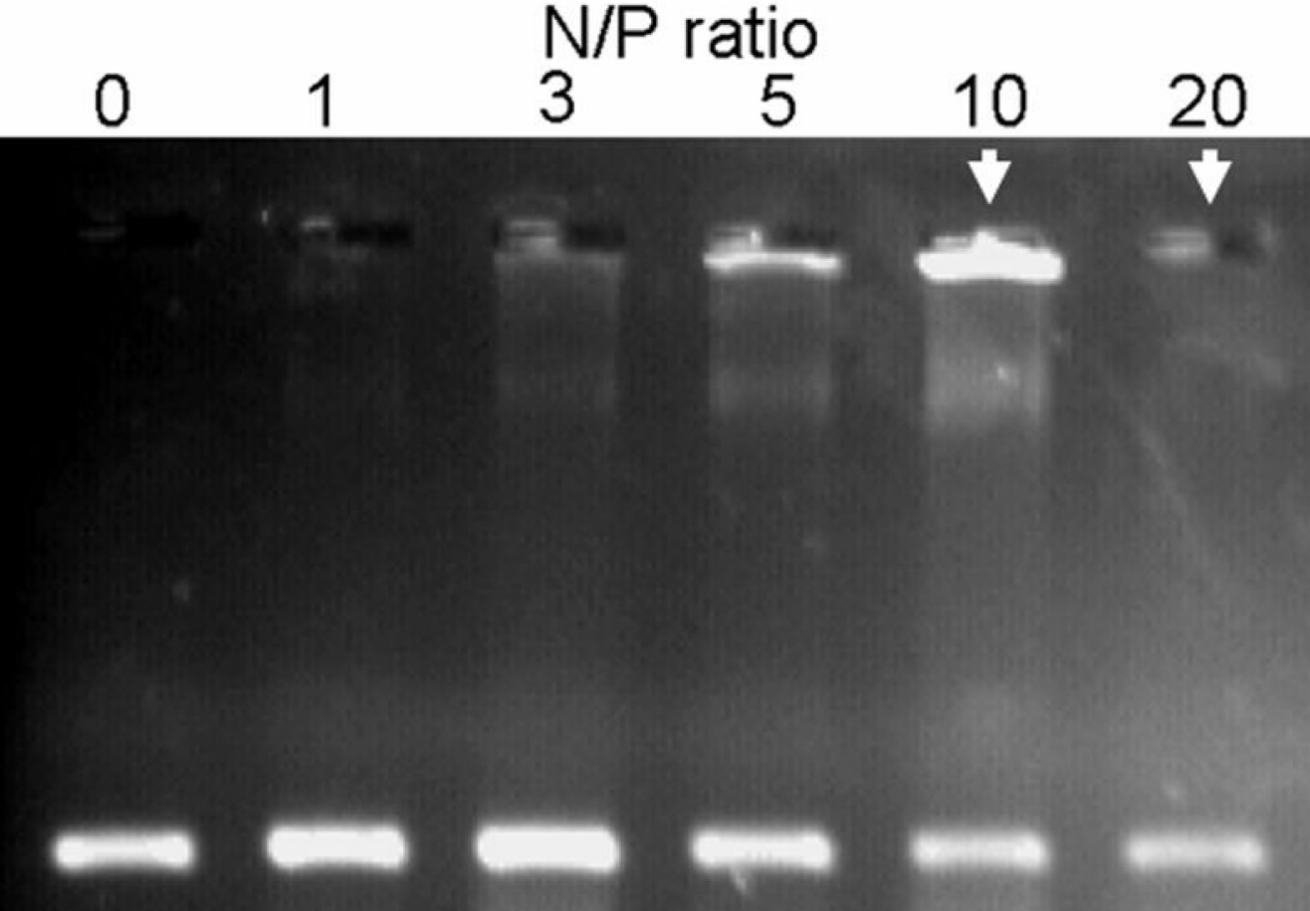
The electrophoretic mobility of CS-*g*-(PEI-*b*-mPEG)/siRNA complexes. The complete retardation is indicated by the white arrow head.

**Figure 5:**
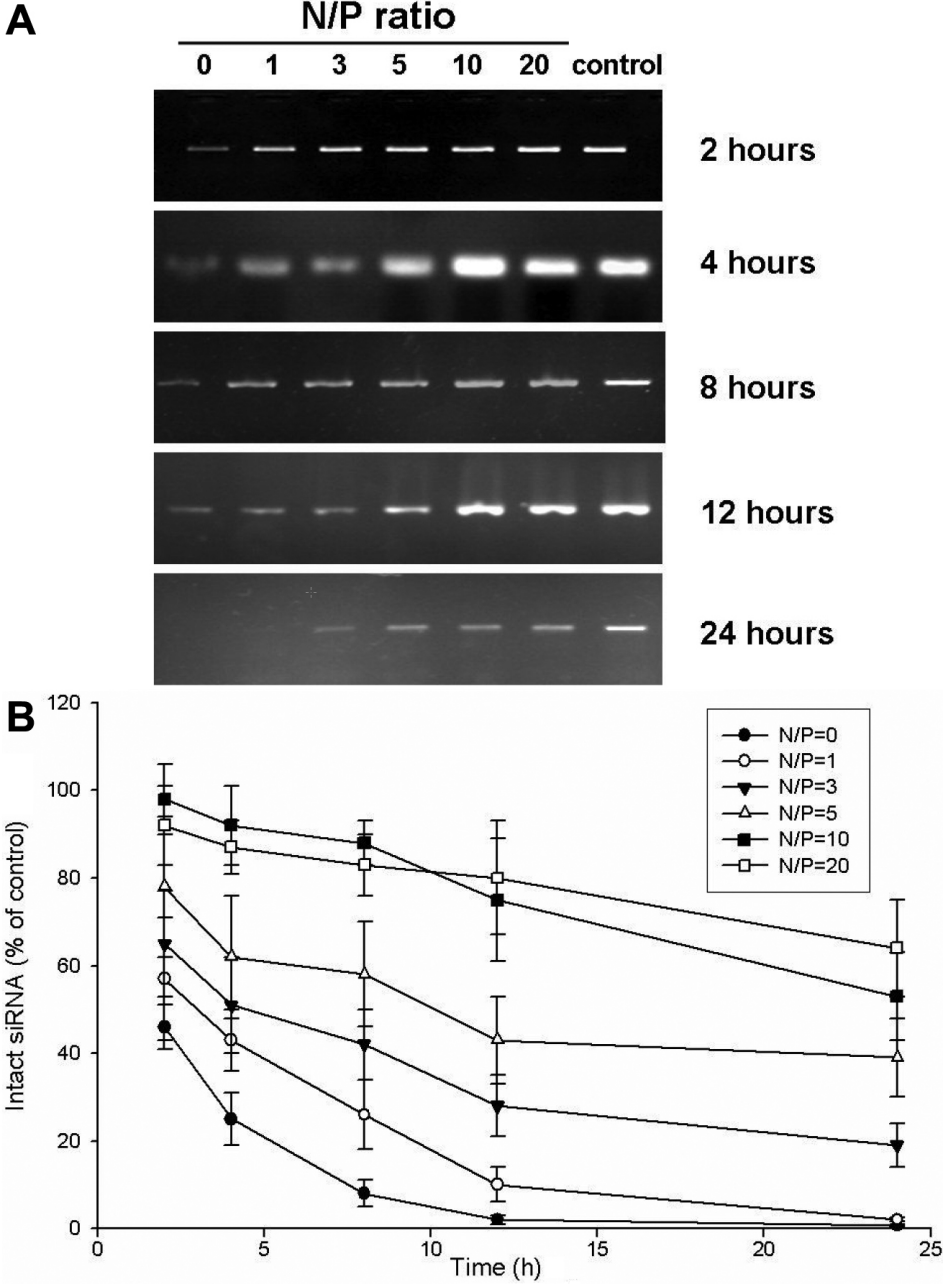
The protection ability of CS-*g*-(PEI-*b*-mPEG) for siRNA against serum degradation. The images of intact siRNAs at determined time intervals are shown (**A**), and the percentage of intact siRNAs in reference to the non-FBS treated control is also calculated (**B**, mean±SD, n=3).

The degree of compaction between CS-*g*-(PEI-*b*-mPEG) and siRNA was assessed by visualizing the mobility of the CS-*g*-(PEI-*b*-mPEG)/siRNA complexes using electrophoresis retardation assay. As shown in Figure 4, the migration of the complexes was retarded to different degrees according to the increasing concentration of the cationic copolymer. Partial retardation was observed at N/P ratios of 1-5, and complete retardation occurred at N/P ratios of 10 and 20. The siRNA protection ability of CS-*g*-(PEI-*b*-mPEG) was measured by incubating the CS-*g*-(PEI-*b*-mPEG)/siRNA complexes with 10% FBS for 2 h, 4 h, 8 h, 12 h, and 24 h. The remaining intact siRNA was visualized by polyacrylamide gel electrophoresis (PAGE), and the images of the gels are shown in Figure 5A. The percentages of siRNAs that were not degraded compared with the control (non-FBS treated siRNAs) are also calculated and shown in Figure 5B.

### Transfection efficiency and cytotoxicity

**Figure 6:**
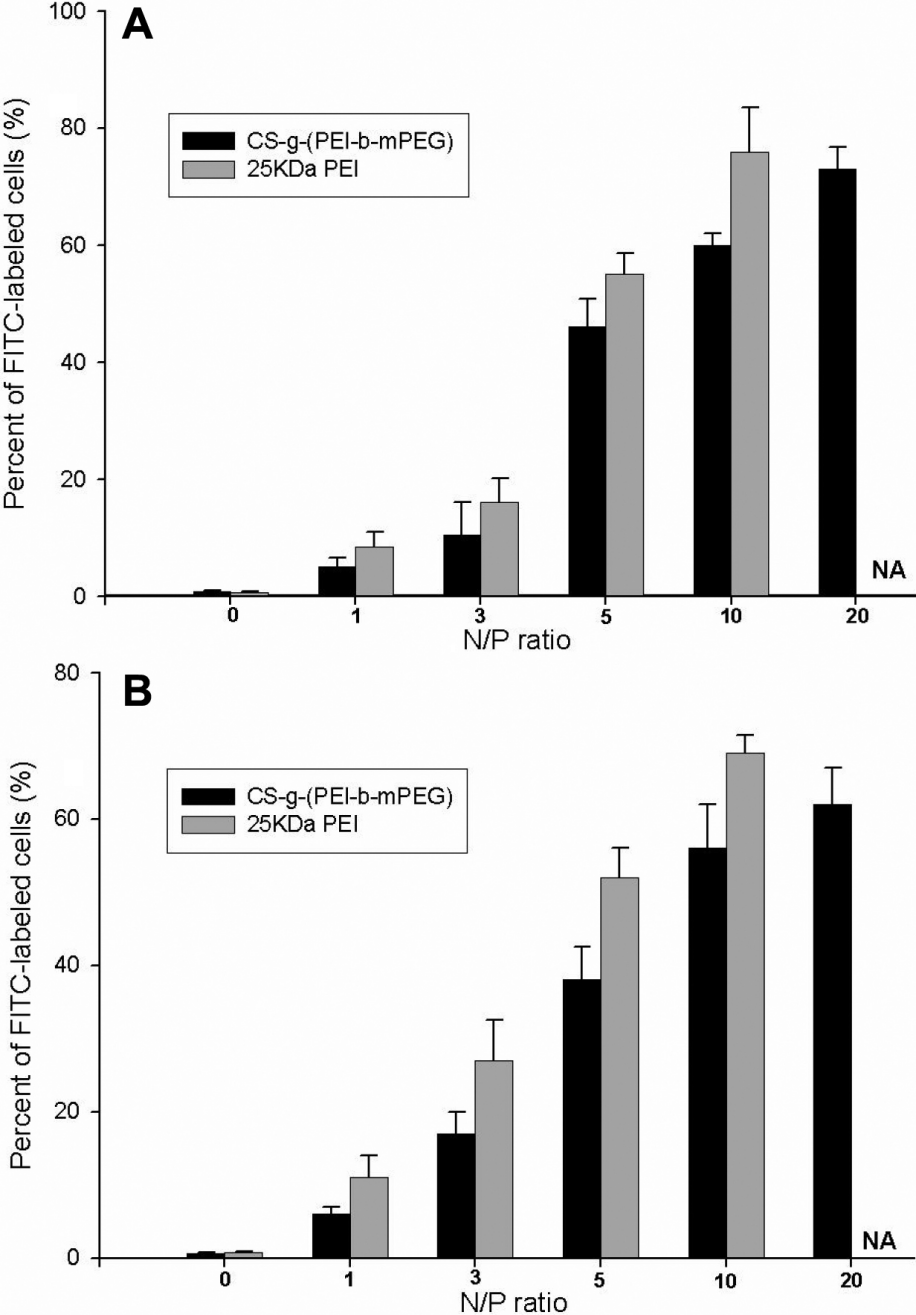
The transfection efficiency of CS-*g*-(PEI-*b*-mPEG) and 25 kDa PEI at different N/P ratios in HeLa cells and HTFs. Data are presented as the percentage of HeLa cells or HTFs containing FITC-conjuncted siRNA, respectively, and data of 25 kDa PEI/siRNA complexes at N/P ratio of 20 can’t be measured (not available, NA). **A**: transfection efficiency in HeLa cells (mean±SD, n=3); **B**: transfection efficiency in HTFs (mean±SD, n=3).

**Figure 7:**
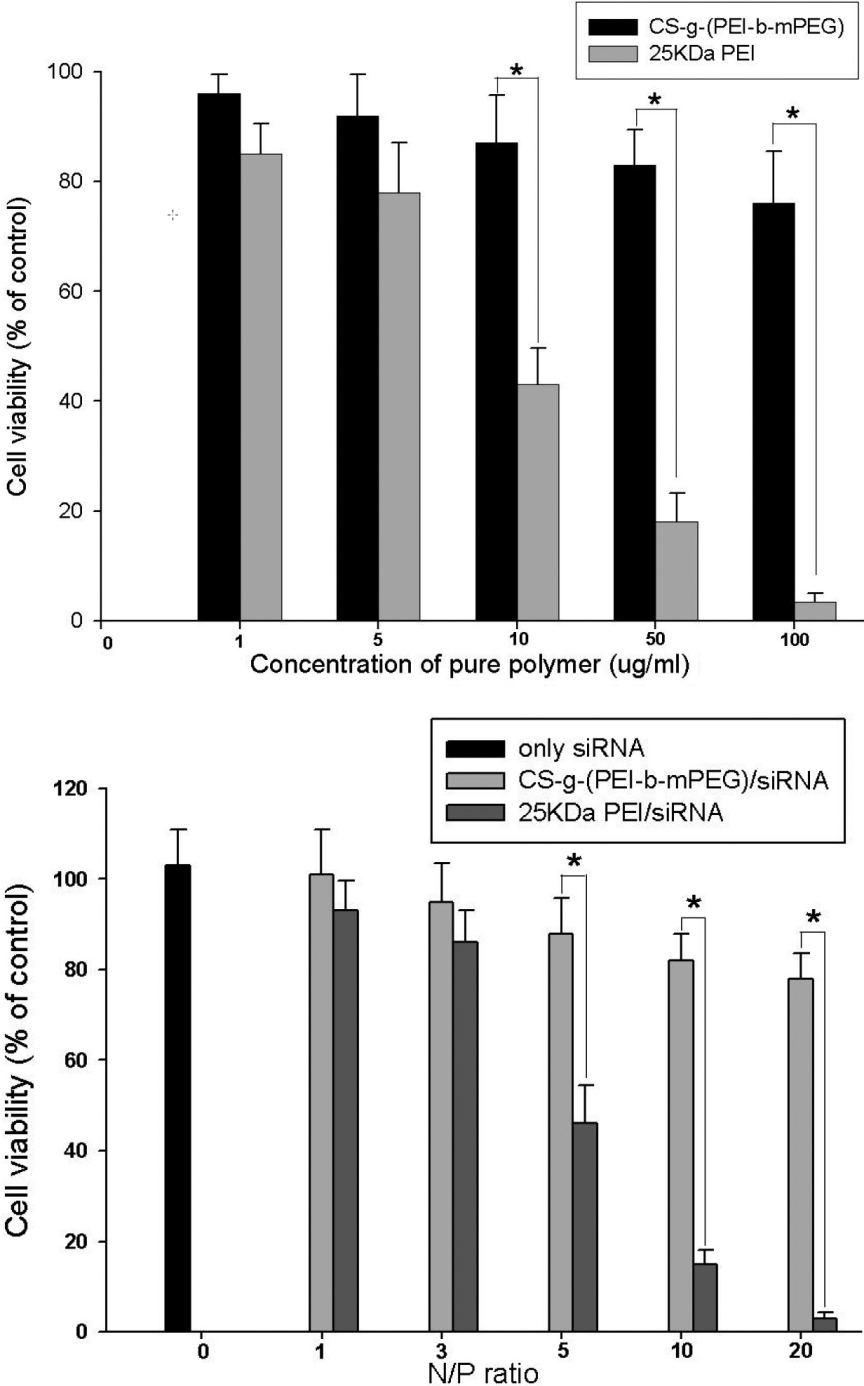
Cytotoxicity of CS-*g*-(PEI-*b*-mPEG) and 25 kDa PEI at various concentrations of pure polymers and complexes with siRNA at determined N/P ratios. Pure CS-*g*-(PEI-*b*-mPEG) copolymer did not exhibit significant cytotoxicity at any determined concentration, and after compacting with siRNAs, both CS-*g*-(PEI-*b*-mPEG)/siRNA and 25 kDa PEI/siRNA complexes showed much lower cytotoxicity. **A**: Cytotoxicity of various concentrations of pure CS-*g*-(PEI-*b*-mPEG) or 25 kDa PEI polymers; **B**: cytotoxicity of CS-*g*-(PEI-*b*-mPEG)/siRNA or 25 kDa PEI/siRNA complexes at determined N/P ratios. Data are presented as the percentage of viable cells compared with the untreated (control) cells (The asterisk indicates a p<0.05, mean ± SD, n=6).

Transfection efficiency was evaluated by measuring the percentage of HeLa cells or HTFs containing FITC-conjugated siRNA, using flowmetry 6 h after CS-*g*-(PEI-*b*-mPEG) or 25 kDa PEI/siRNA complexes were added. As shown in Figure 6, both the transfection efficiency of CS-*g*-(PEI-*b*-mPEG) and 25 kDa PEI increased as the N/P ratio increased. The transfection efficiency of CS-*g*-(PEI-*b*-mPEG) reached its peak (more than 60%) at the N/P ratio of 20 in both cell lines. However, it could not be calculated when 25 kDa PEI was used for transfection at the same N/P ratio because most cells had been killed. A tetrazolium-based viability assay, which was based on the bioreduction of the MTS reagent into formazan by living cells, was used to study the cytotoxicity of CS-*g*-(PEI-*b*-mPEG) and 25 kDa PEI. The pure CS-*g*-(PEI-*b*-mPEG) copolymer exhibited cell viability of more than 70% at concentrations of 1–100 μg/ml, and the number of viable cells was significantly higher than those treated with pure 25 kDa PEI at concentrations of 10 μg/ml and above (Figure 7A). Figure 7B reveals that both the CS-*g*-(PEI-*b*-mPEG)/siRNA and 25 kDa PEI/siRNA complexes presented less toxicity after being compacted with siRNAs, and the CS-*g*-(PEI-*b*-mPEG)/siRNA complexes also performed decreasing cytotoxicity at all the determined N/P ratios compared with that of the 25 kDa PEI/siRNA complexes.

### Downregulating effect on *IKKβ* expression

**Figure 8:**
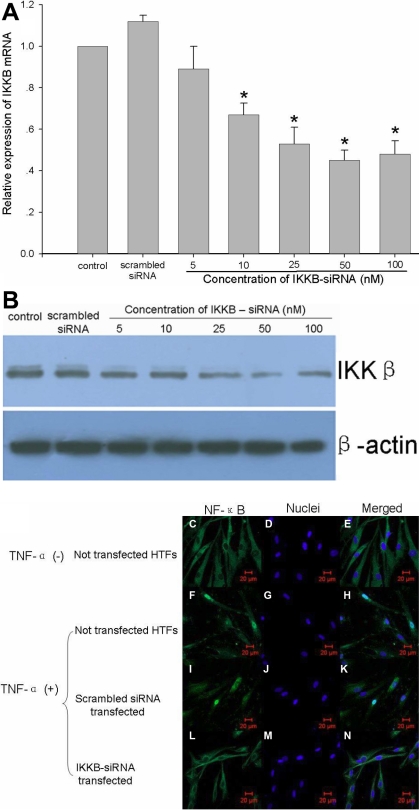
IKKΒ-siRNA inhibits the expression of *IKKβ* on both the mRNA and protein level. **A**: mRNA transcription of *IKKβ* in HTFs assessed by real-time RT-PCR 24 h after 5-100 nM IKKΒ-siRNA was transfected. The normalized *IKKβ* mRNA level of non-transfected HTFs is taken as 1.0 (the asterisk indicates a p<0.05, mean±SD, n=3). **B**: Protein levels of IKKβ demonstrated by western blot. **C**-**N**: Confocal laser scanning microscopy images shows the intracellular distribution of NF-κB in HTFs. Green fluorescence indicates the intracellular distribution of phosphated NF-κB, and blue fluorescence represents the DAPI counterstained cell nuclei.

In our primary experiments, we found that IKKΒ-si1 is more effective than IKKΒ-si2 in downregulating the transcription of *IKKβ* mRNA (data not shown), so we used IKKΒ-si1 as IKKΒ-siRNA in the subsequent RNAi procedures. Real-time PCR assay revealed that mRNA transcription of *IKKβ* in the HTFs was suppressed in a dose-dependent manner 24 h after 5–100 nM of IKKΒ-siRNA were transfected (Figure 8A). Significant inhibition (43%) was detected following transfection of 10 nM of IKKΒ-siRNA compared to the control group (p<0.05), and maximum suppression (55%) was observed in the group transfected with 50 nM of IKKΒ-siRNA. In addition, no significant difference between the mRNA level of *IKKβ* was detected in multiple experiments following 50 nM and 100 nM of IKKΒ-siRNA transfection, and cells transfected with 100 nM of scrambled siRNA expressed a level of *IKKβ* mRNA similar to that of the control group. Meanwhile, the IKKβ protein level was demonstrated by western blot assay 48 h after 5–100 nM of IKKΒ-siRNA were transfected into the HTFs. The expression of IKKβ protein was inhibited in a dose-dependent manner after IKKΒ-siRNA transfection into the HTFs whereas no significant difference was found between the expression level of β-actin in HTFs that were and were not transfected (Figure 8B).

### Inhibiting effect on the activation of NF-κB

The activation of NF-κB was determined by immunocytochemical imaging. Immunofluorescence detected by confocal laser scan microscopy showed that activated NF-κB were present in both the cytoplasm and nuclei of normal HTFs (Figure 8C-E), and after stimulation with TNF-α, most translocated into the nuclei (Figure 8F-H). After transfection with 100 nM of IKKΒ-siRNA for 72 h, no more activated NF-κB could be seen in the nuclei of the HTFs after the stimulation with TNF-α whereas many were still present in the cytoplasm (Figure 8L-N). In contrast, inhibition of the activation and translocation of NF-κB was not found in HTFs transfected with 100 nM of scrambled siRNA (Figure 8I-K).

### Inhibiting the proliferation of human Tenon’s capsule fibroblasts by RNAi targeting *IKKβ*

**Figure 9:**
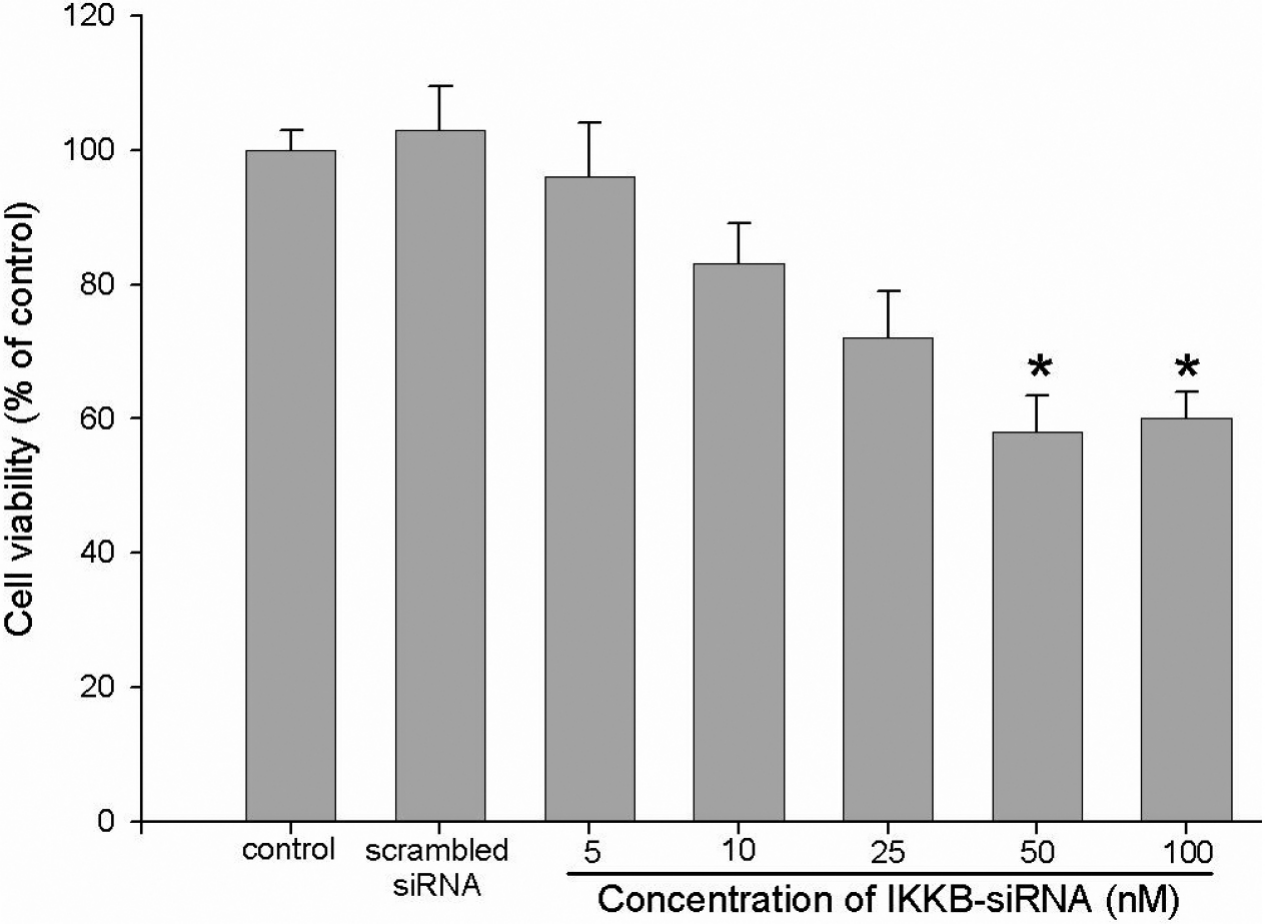
The inhibition effect of blocking NF-κB pathway on the proliferation of HTFs through RNAi. Data are presented as the percentage of viable cells compared with the untreated (control) cells (mean ± SD, n=6). An asterisk indicates that p<0.05.

The RNAi process targeting *IKKβ* repressed the proliferation of HTFs in a siRNA dose-dependent manner in vitro. The cell viability of HTFs transfected with more than 50 nM of IKKΒ-siRNA showed significant differences compared with that of the control group (p<0.05) as shown in Figure 9 whereas the proliferation of HTFs transfected with 100 nM of scrambled siRNA was not affected.

## Discussion

Glaucoma filtration surgery often fails because of excessive scarring that occurs during the wound healing process. After filtration surgery, fibroblasts are activated by various cytokines and growth factors, eventually resulting in the closure of the drainage channel. Thus, preventing the hyperfunction of fibroblasts is an important strategy for minimizing subconjunctival scarring [20,21]. Current anti-scarring therapies focus on suppressing the proliferation of fibroblasts through anti-metabolic agents such as mitomycin C (MMC) and 5-fluorouracil (5-Fu). The perioperative administration of such agents has greatly improved the success rate of filtration surgery [22,23]. However, they are associated with several severe complications including keratitis, bleb leakage, chronic hypotony with maculopathy, and endophthalmitis [24].

One goal of our study was to find new physiologic approaches to limiting excessive subconjunctival wound healing that are as effective as antimetabolic agents but have fewer adverse effects. NF-κB is a member of the transcription factor family NF-κB/Rel and was originally described as a necessary element for the transcription of the immunoglobulin κL chain gene in mature B cells [25]. NF-κB has recently proved to be a ubiquitous factor associated with wound healing through the factor’s ability to stimulate transcription of various genes involved in the activation of inflammation and cell proliferation [26,27]. Thus, we thought the NF-κB pathway may be an essential factor in the regulation of the proliferation of HTFs after glaucoma filtration surgery. NF-κB is bonded with an inhibitor protein, IκB, which sequesters NF-κB in an inactive form in the cytoplasm. A specific IκB kinase, IKK, is a protein complex that contains three subunits, and studies indicate that IKKβ is indispensable for the activation of NF-κB [28]. IKK phosphorylates IκB and initiates the inhibitor’s conjugation to ubiquitin and subsequent degradation by proteasomes. In turn, NF-κB is activated through release from IκB and translocates into the nuclei [29]. RNAi is commonly used as a powerful tool in biological and biomedical research [30] and has been used experimentally to prevent ocular neovascularization and inflammation [31,32]. Researchers in our group have successfully inhibited the proliferation of HTFs through specifically downregulating the expression of *IKKβ* using a commercial transfection reagent, Lipofectamine^TM^ 2000 (Invitrogen, Carlsbad, CA), as a siRNA-delivering vector. However, this cationic liposome compound cannot be used for in vivo investigation in humans [33].

Therefore, another objective of our study was to design and synthesize new compounds that are non-toxic, non-immunogenic, degradable, and efficient for delivering siRNA into HTFs. Among non-viral nucleotide carriers currently under investigation, PEI is one kind of synthetic polymer with a high cationic charge density and a protonable amino group in every third position. PEI can condense and compact DNA into complexes, and the strong proton capacity of PEI allows it to deliver plasmid DNA and oligonucleotides into mammalian cells, both in vitro and in vivo [34]. However, despite having effective delivery capacity, high molecular weight PEI can not be degraded by body fluids and its high cationic charge density also makes PEI toxic to cells [35]. Therefore, the current trend is toward using modified low molecular weight PEI as a nucleotide delivery reagent, which combines high biocompatibility and reduced cytotoxicity [36,37]. PEG, a nonionic hydrophilic polyether, has been widely investigated as a synthesizing graft copolymer with PEI. The nonionic PEG chains can serve as a hydrophilic shell, which makes the new copolymer more soluble and stable, and reduces its non-specific interaction with proteins in physiologic fluids [38,39]. One drawback of these copolymers for in vivo application is their lack of transfection efficiency. Chitosan is the name given to a group of linear cationic polymers of glucosamine and N-acetylglucosamine that are derived from the natural biopolymer, chitin, by alkaline deacetylation. Chitosan has been investigated as a food additive or a wound dressing, and it has been recently considered to be a good candidate for gene delivery because of its reported biocompatibility and biodegradability and its relatively non-toxic nature [40].

In light of these results, we speculated that a novel cationic copolymer combined with PEI, PEG, and CS could be synthesized as a vehicle for delivering siRNA into mammalian cells effectively and safely. In our research, we found that after the grafting reaction, characteristic absorption of PEI and mPEG appeared at 2.5−3.3 ppm and 3.5 ppm in the ^1^H NMR spectrum of the copolymer, respectively. CS proton absorption signals (H-2, H-3, 4, 5, 6, 6’, 2.8−3.8 ppm) also overlapped the PEI and mPEG proton signals (Figure 2). All of the data above prove that the designed CS-*g*-(PEI-*b*-mPEG) copolymer was successfully obtained. The molecular weight of CS-*g*-(PEI-*b*-mPEG) was 2.95×10^4^, which was relatively larger than some reported PEI-*alt*-PEG copolymers [41] but smaller than generally used PEI [37].

As indicated in previous reports, the size and shape of nanoparticles play an important role in the delivery process and greatly influence distribution in the body [42]. It has been reported that the nano-size of particles is a key prerequisite for cell uptake [43]. Similar to plasmid DNA and oligonucleotides, siRNAs are taken up by cells through endocytosis. Therefore, suitable particle size has an important influence on the delivering capacity of a siRNA vector. We investigated the diameter of the CS-*g*-(PEI-*b*-mPEG)/siRNA complexes at five different N/P charge ratios and observed that the complexes represented particles with nanometer size (about 200 nm), which were much less than that of 25 kDa PEI. Furthermore, particle size tended to decrease as the N/P charge ratios of the CS-*g*-(PEI-*b*-mPEG)/siRNA complexes increased, indicating that the CS-*g*-(PEI-*b*-mPEG) copolymer can condense siRNA into a more compact structure, mainly owing to the net electrostatic repulsive forces between complexes.

The surface charge of the CS-*g*-(PEI-*b*-mPEG)/siRNA complexes is also a major factor influencing transfection efficiency. After the compaction between cationic copolymers and siRNA, the negative charge of siRNA is neutralized and the newly assembled nanoparticles may retain a partial positive surface charge to help siRNA pass through the cell membrane and escape the endolysosomes. However, the excessive positive charge of PEI homopolymers may subsequently lead to hyperpermeability of the membrane, resulting in cell death [44]. The density of the surface charge is reflected by measured zeta potential values, and as we have shown, an initial negative value of zeta potential (−2.4 mV) was detected when the complexes formed at an N/P ratio of 1, which means that siRNA could not be completely compacted under this condition. Then, the positive surface charge of the complexes exhibited an increasing trend corresponding to the rising N/P ratio, indicating that more and more siRNAs were compacted with CS-*g*-(PEI-*b*-mPEG) and the negative charge was neutralized. But the zeta potential of the CS-*g*-(PEI-*b*-mPEG)/siRNA complexes at each N/P ratio that we measured was still lower than that of the 25 kDa PEI/siRNA complexes. The reason for this result is probably consistent with the reason Petersen et al. [8] regarding copolymer-based DNA complexes, which was that the shielding effect of neutral components on the PEI part of the copolymer produces a relatively low zeta potential. 

Condensation ability is one requirement for a siRNA carrier. An optimal binding degree between CS-*g*-(PEI-*b*-mPEG) and siRNA can achieve more efficient delivering capacity. If the complexes are formed efficiently, all siRNAs are bound to the copolymers to form nanoparticles. Hence, the complexes become relatively large and remain immobile in the loading well with no bands of free siRNA apparent. We observed that the migration of siRNA was retarded to different degrees in accordance with the increasing N/P ratio. Complete retardation occurs at an N/P ratio of 10, which means the CS-*g*-(PEI-*b*-mPEG)/siRNA complexes are completely formed. The complete complexes of 600 kDa PEI and siRNA cannot be found even at an N/P ratio of 50 (data not shown), which indicates that the condensation ability of CS-*g*-(PEI-*b*-mPEG) is better than low molecular weight PEI. It was also detected that the band of siRNA at an N/P ratio of 20 demonstrated much lower fluorescence intensity than that of the band at the N/P ratio of 10. This decrease in fluorescence was also observed by other experiments executed on DNA bands [8,45]. The reason for this phenomenon is that the measured fluorescence is attributed to the intercalated ethidium bromide in siRNA. When the condensation degree between siRNA and the cationic copolymer gradually increases, ethidium bromide cannot intercalate with siRNA anymore. Therefore, the fluorescence intensity of the bands decreases accordingly [39].

The main hindrance to the use of RNAi as a therapeutic tool for human diseases is that the unprotected dsRNA or siRNA will be rapidly degraded by either nucleases in serum or the endosomal compartment of cells. The enzymatic degradation of siRNA is accompanied by a rapid decline in biological activity and therapeutic efficiency. Therefore, the potential of this technology as a clinical therapy method depends largely on the improvement of siRNA vectors’ protection ability against enzymatic degradation. We found that after incubation in 10% FBS for 24 h, 64% of siRNA was protected from degradation at the N/P ratio of 20 whereas only 0.7% naked siRNA remained intact. We attributed these results to two factors. First, PEG not only has the reported ability to stabilize the structure of nanospheres, but PEG can also protect siRNA from being attacked by nucleases [46,47]. Second, it has been reported that chitosan can effectively protect DNA from nuclease degradation [48]. The CS-*g*-(PEI-*b*-mPEG) we have synthesized has both PEG and CS elements and can provide efficient protection for siRNA against enzymatic degradation.

Low toxicity is also a major requirement for an siRNA delivery system. The cytotoxicity of cationic copolymers is mainly caused by the aggregation of nanoparticles on the cell membrane, impairing its normal function. In addition, the excessive positive surface charge of nanoparticles may also interfere with critical intracellular processes of cells [44]. It has been shown that chitosan salts and chitosan derivatives are less toxic than PEI [49]. However, no data have been reported regarding the cytotoxic analysis of a synthetic cationic copolymer on human Tenon’s capsule fibroblasts. Therefore, we explored the influence of different concentrations of CS-*g*-(PEI-*b*-mPEG) and 25 kDa PEI on the cell viability of HTFs. CS-*g*-(PEI-*b*-mPEG) showed much less cytotoxicity than 25 kDa PEI, which is consistent with what had been obtained by Kim et al. [18] in tests of HeLa and HepG2 cells. The cell viabilities of the HTFs decreased drastically as the concentrations of 25 kDa PEI increased whereas the pure CS-*g*-(PEI-*b*-mPEG) copolymer did not exhibit significant cytotoxicity at any determined concentration. We hypothesize that the relatively low cytotoxicity of CS-*g*-(PEI-*b*-mPEG) could be explained from two aspects. First, the copolymer can be degraded into CS, PEG, and low molecular weight PEI units in cells, all of which can be easily eliminated by excretion pathways, thus making this copolymer relatively less toxic than 25 kDa PEI. Second, PEG reduces toxicity by substituting the amino groups of PEI, which are the main toxic moieties of the copolymer [50]. Furthermore, after compacting with siRNA, both the CS-*g*-(PEI-*b*-mPEG)/siRNA and 25 kDa PEI/siRNA complexes showed much lower cytotoxicity, which is due to the neutralization effect of the negative charge of siRNA on the positive charge of the pure polymers.

The transfection efficiency of the CS-*g*-(PEI-*b*-mPEG)/siRNA complexes was assessed at various N/P ratios in HeLa cells and HTFs. This is mainly because the N/P ratio is directly related to the size, surface charge, compaction degree, and serum-resistant capacity of the CS-*g*-(PEI-*b*-mPEG)/siRNA complexes, all of which can affect delivery efficiency. At the N/P ratio of 20, CS-*g*-(PEI-*b*-mPEG) outperformed the highest transfection rate in both HeLa cells and HTFs, which can be explained by the appropriate particle size and surface charge as well as the excellent stability of the CS-*g*-(PEI-*b*-mPEG)/siRNA complexes. The transfection rate was a little lower than that of 25 kDa PEI at the same N/P ratio partly because of the shielding effect of mPEG on the positive charge of PEI. We also found that both CS-*g*-(PEI-*b*-mPEG) and 25 kDa PEI showed a lower transfection rate in HTFs than in HeLa cells, which may be attributed to a cell line dependency of the cationic polymer’s delivery ability [51]. From our results, we can conclude that PEI with low molecular weight grafted onto CS avoids the cytotoxicity of high molecular weight PEI. Meanwhile, mPEG improves the stability of CS-*g*-(PEI-*b*-mPEG)/siRNA complexes, and as a result, the CS-*g*-(PEI-*b*-mPEG)/siRNA nanoparticles can offer a substantial gene silencing effect with minimal side effects. Moreover, many factors governing the transfection efficiency of cationic copolymers need to be investigated in future studies such as the presence of serum and the pH value of solution [52], and ligands will be conjugated to the copolymers to achieve receptor-mediated endocytosis and potentially to target cells or tissues.

A special siRNA targeting *IKKβ* gene was successfully compacted with CS-*g*-(PEI-*b*-mPEG) and effectively delivered into HTFs. Subsequently, both the mRNA and protein levels of *IKKβ* were suppressed, and the activation of NF-κB was inhibited in turn. Finally, IKKΒ-siRNA-mediated blocking of the NF-κB pathway resulted in repression of the proliferation of HTFs, and cells transfected with IKKΒ-siRNA showed growth inhibition up to 42%. All of these findings suggest that blocking the signaling pathway of NF-κB could be an effective way to manipulate the scar formation process by downregulating the proliferation of HTFs. This novel method based on nanotechnology and RNAi could represent a remarkable anti-scarring therapeutic approach for glaucoma filtration surgery. Follow-up studies will focus on the in vivo application of CS-*g*-(PEI-*b*-mPEG)/IKKΒ-siRNA complexes.

We have reported on the synthesis and characterization of a novel cationic copolymer, CS-*g*-(PEI-*b*-mPEG). We found it to have powerful siRNA binding and protection ability, relatively high transfection efficiency, and low cytotoxicity, making CS-*g*-(PEI-*b*-mPEG) a suitable delivery vector for transfecting siRNA into cells. We observed that siRNA targeting *IKKβ* was successfully transfected into human Tenon’s capsule fibroblasts in vitro, and RNAi processes against the expression of *IKKβ* can subsequently inhibit the activation of NF-κB and in turn, the proliferation of HTFs. Our results indicate the potential for a safe and effective strategy for preventing scar formation after glaucoma filtration surgery.

## References

[1] Plasterk RH (2002). RNA silencing: the genome's immune system.. Science.

[2] Hannon GJ (2002). RNA interference.. Nature.

[3] Elbashir SM, Harborth J, Lendeckel W, Yalcin A, Weber K, Tuschl T (2001). Duplexes of 21-nucleotide RNAs mediate RNA interference in cultured mammalian cells.. Nature.

[4] Sun JY, Anand-Jawa V, Chatterjee S, Wong KK (2003). Immune responses to adeno-associated virus and its recombinant vectors.. Gene Ther.

[5] Lehrman S (1999). Virus treatment questioned after gene therapy death.. Nature.

[6] Merdan T, Kopecek J, Kissel T (2002). Prospects for cationic polymers in gene and oligonucleotide therapy against cancer.. Adv Drug Deliv Rev.

[7] Boussif O (1995). Lezoualc'h F, Zanta MA, Mergny MD, Scherman D, Demeneix B, Behr JP. A versatile vector for gene and oligonucleotide transfer into cells in culture and in vivo: polyethylenimine.. Proc Natl Acad Sci USA.

[8] Petersen H, Fechner PM, Martin AL, Kunath K, Stolnik S, Roberts CJ, Fischer D, Davies MC, Kissel T (2002). Polyethylenimine-graft-poly (ethylene glycol) copolymers: influence of copolymer block structure on DNA complexation and biological activities as gene delivery system.. Bioconjug Chem.

[9] Ahn CH, Chae SY, Bae YH, Kim SW (2002). Biodegradable poly(ethylenimine) for plasmid DNA delivery.. J Control Release.

[10] Katas H, Alpar HO (2006). Development and characterisation of chitosan nanoparticles for siRNA delivery.. J Control Release.

[11] The Advanced Glaucoma Intervention Study (AGIS):7. The relationship between control of intraocular pressure and visual field deterioration.The AGIS Investigators. Am J Ophthalmol 2000130429 40 11024415 10.1016/s0002-9394(00)00538-9

[12] Cordeiro MF, Chang L, Lim KS, Daniels JT, Pleass RD, Siriwardena D, Khaw PT (2000). Modulating conjunctival wound healing.. Eye.

[13] Khaw PT, Occleston NL, Schultz G, Grierson I, Sherwood MB, Larkin G (1994). Activation and suppression of fibroblast function.. Eye.

[14] Kessler DJ, Duyao MP, Spicer DB, Sonenshein GE (1992). NF-kappa B-like factors mediate interleukin 1 induction of c-myc gene transcription in fibroblasts.. J Exp Med.

[15] DiDonato JA, Hayakawa M, Rothwarf DM, Zandi E, Karin M (1997). A cytokine-responsive IkappaB kinase that activates the transcription factor NF-kappaB.. Nature.

[16] Khaw PT, Ward S, Porter A, Grierson I, Hitchings RA, Rice NS (1992). The long-term effects of 5-fluorouracil and sodium butyrate on human Tenon's fibroblasts.. Invest Ophthalmol Vis Sci.

[17] Jiang HL, Kim YK, Arote R, Nah JW, Cho MH, Choi YJ, Akaike T, Cho CS (2007). Chitosan-graft-polyethylenimine as a gene carrier.. J Control Release.

[18] Kim TH, Park IK, Nah JW, Choi YJ, Cho CS (2004). Galactosylated chitosan/DNA nanoparticles prepared using water-soluble chitosan as a gene carrier.. Biomaterials.

[19] Livak KJ, Schmittgen TD (2001). Analysis of relative gene expression data using real-time quantitative PCR and the 2(-Delta Delta C (T)).. Methods.

[20] Khaw PT, Chang L, Wong TT, Mead A, Daniels JT, Cordeiro MF (2001). Modulation of wound healing after glaucoma surgery.. Curr Opin Ophthalmol.

[21] Atreides SP, Skuta GL, Reynolds AC (2004). Wound healing modulation in glaucoma filtering surgery.. Int Ophthalmol Clin.

[22] Smith MF, Doyle JW, Nguyen QH, Sherwood MB (1997). Results of intraoperative 5-fluorouracil or lower dose mitomycin-C administration on initial trabeculectomy surgery.. J Glaucoma.

[23] Singh RP, Goldberg I, Mohsin M (2001). The efficacy and safety of intraoperative and/or postoperative 5-fluorouracil in trabeculectomy and phacotrabeculectomy.. Clin Experiment Ophthalmol.

[24] Sihota R, Dada T, Gupta SD, Sharma S, Arora R, Agarwal HC (2000). Conjunctival dysfunction and mitomycin C-induced hypotony.. J Glaucoma.

[25] Baeuerle PA, Baltimore D (1996). NF-kappa B: ten years after.. Cell.

[26] Kaltschmidt B, Kaltschmidt C, Hehner SP, Droge W, Schmitz ML (1999). Repression of NF-kappaB impairs HeLa cell proliferation by functional interference with cell cycle checkpoint regulators.. Oncogene.

[27] Li M, Carpio DF, Zheng Y, Bruzzo P, Singh V, Ouaaz F, Medzhitov RM, Beg AA (2001). An essential role of the NF-kappa B/Toll-like receptor pathway in induction of inflammatory and tissue-repair gene expression by necrotic cells.. J Immunol.

[28] Zandi E, Rothwarf DM, Delhase M, Hayakawa M, Karin M (1997). The IkappaB kinase complex (IKK) contains two kinase subunits, IKKalpha and IKKbeta, necessary for IkappaB phosphorylation and NF-kappaB activation.. Cell.

[29] Woronicz JD, Gao X, Cao Z, Rothe M, Goeddel DV (1997). IkappaB kinase-beta: NF-kappaB activation and complex formation with IkappaB kinase-alpha and NIK.. Science.

[30] Novina CD, Sharp PA (2004). The RNAi revolution.. Nature.

[31] Reich SJ, Fosnot J, Kuroki A, Tang W, Yang X, Maguire AM, Bennett J, Tolentino MJ (2003). Small interfering RNA (siRNA) targeting VEGF effectively inhibits ocular neovascularization in a mouse model.. Mol Vis.

[32] Nakamura H, Siddiqui SS, Shen X, Malik AB, Pulido JS, Kumar NM, Yue BY (2004). RNA interference targeting transforming growth factor-beta type II receptor suppresses ocular inflammation and fibrosis.. Mol Vis.

[33] Huang SS, Ge J, Wang LN, Yin XB, Wei YT, Ma P (2005). Preliminary study of inhibition of human Tenon’s capsule fibroblasts in vitro by RNA interference targeting IKK-beta.. Zhonghua Yan Ke Za Zhi.

[34] Kichler A, Leborgne C, Coeytaux E, Danos O (2001). Polyethylenimine-mediated gene delivery: a mechanistic study.. J Gene Med.

[35] Moghimi SM, Symonds P, Murray JC, Hunter AC, Debska G, Szewczyk A (2005). A two-stage poly (ethylenimine)-mediated cytotoxicity: implications for gene transfer/therapy.. Mol Ther.

[36] Kim YH, Park JH, Lee M, Kim YH, Park TG, Kim SW (2005). Polyethylenimine with acid-labile linkages as a biodegradable gene carrier.. J Control Release.

[37] Fischer D, Bieber T, Li Y, Elsasser HP, Kissel T (1999). A novel non-viral vector for DNA delivery based on low molecular weight, branched polyethylenimine: effect of molecular weight on transfection efficiency and cytotoxicity.. Pharm Res.

[38] Brus C, Petersen H, Aigner A, Czubayko F, Kissel T (2004). Physicochemical and biological characterization of polyethylenimine-graft-poly (ethylene glycol) block copolymers as a delivery system for oligonucleotides and ribozymes.. Bioconjug Chem.

[39] Mao S, Neu M, Germershaus O, Merkel O, Sitterberg J, Bakowsky U, Kissel T (2006). Influence of polyethylene glycol chain length on the physicochemical and biological properties of poly(ethylene imine)-graft-poly(ethylene glycol) block copolymer/siRNA polyplexes.. Bioconjug Chem.

[40] Mansouri S, Cuie Y, Winnik F, Shi Q, Lavigne P, Benderdour M, Beaumont E, Fernandes JC (2006). Characterization of folate-chitosan-DNA nanoparticles for gene therapy.. Biomaterials.

[41] Park MR, Han KO, Han IK, Cho MH, Nah JW, Choi YJ, Cho CS (2005). Degradable polyethylenimine-alt-poly (ethylene glycol) copolymers as novel gene carriers.. J Control Release.

[42] Schiffelers RM, Woodle MC, Scaria P (2004). Pharmaceutical prospects for RNA interference.. Pharm Res.

[43] Liu G, Molas M, Grossmann GA, Pasumarthy M, Perales JC, Cooper MJ, Hanson RW (2001). Biological properties of poly-L-lysine-DNA complexes generated by cooperative binding of the polycation.. J Biol Chem.

[44] Fischer D, Li Y, Ahlemeyer B, Krieglstein J, Kissel T (2003). In vitro cytotoxicity testing of polycations: influence of polymer structure on cell viability and hemolysis.. Biomaterials.

[45] Kim TH, Kim SI, Akaike T, Cho CS (2005). Synergistic effect of poly (ethylenimine) on the transfection efficiency of galactosylated chitosan/DNA complexes.. J Control Release.

[46] Kim SH, Jeong JH, Lee SH, Kim SW, Park TG (2006). PEG conjugated VEGF siRNA for anti-angiogenic gene therapy.. J Control Release.

[47] Lee SH, Kim SH, Park TG (2007). Intracellular siRNA delivery system using polyelectrolyte complex micelles prepared from VEGF siRNA-PEG conjugate and cationic fusogenic peptide.. Biochem Biophys Res Commun.

[48] Corsi K, Chellat F, Yahia L, Fernandes JC (2003). Mesenchymal stem cells, MG63 and HEK293 transfection using chitosan-DNA nanoparticles.. Biomaterials.

[49] Thanou M, Florea BI, Geldof M, Junginger HE, Borchard G (2002). Quaternized chitosan oligomers as novel gene delivery vectors in epithelial cell lines.. Biomaterials.

[50] Kostarelos K (2003). Rational design and engineering of delivery systems for therapeutics: biomedical exercises in colloid and surface science.. Adv Colloid Interface Sci.

[51] Florea BI, Meaney C, Junginger HE, Borchard G (2002). Transfection efficiency and toxicity of polyethylenimine in differentiated Calu-3 and nondifferentiated COS-1 cell cultures.. AAPS PharmSci.

[52] Sato T, Ishii T, Okahata Y (2001). In vitro gene delivery mediated by chitosan. Effect of pH, serum, and molecular mass of chitosan on the transfection efficiency.. Biomaterials.

